# Looking Beyond *Leishmania infantum*: Serological and Molecular Investigation of *Leishmania* spp. in Domestic Dogs and Cats from a Visceral Leishmaniasis-Endemic Area in Northeastern Brazil

**DOI:** 10.1007/s11686-026-01408-9

**Published:** 2026-09-26

**Authors:** Ana Vitória Verde Oliveira Rocha, Thaliane França Costa, Renata Mondêgo de Oliveira, Felipe Trovalim Jordão, Marcia Aparecida Speranca, Marcia Dalastra Laurenti, Aline Diniz Cabral, Francisco Borges Costa, Rita de Maria Seabra Nogueira, Andréa Pereira da Costa

**Affiliations:** 1 State University of Maranhão, Av. Lourenço Vieira da Silva, 1000, São Luís, Maranhão 65055-310 Brazil; 2 Federal University of ABC (UFABC), Av. Dos Estados, 5001, Bangu, Santo André, São Paulo 09210-580 Brazil; 3 Faculty of Medicine, University of São Paulo, Av. Dr. Arnaldo, 455, São Paulo, 01246-903 Brazil

**Keywords:** Seroreactivity, PCR–RFLP, Molecular epidemiology, Peri-urban environment, Domestic animal infection

## Abstract

**Purpose:**

The urbanization of zoonotic leishmaniasis and the potential overlap of transmission cycles involving different *Leishmania* species, vertebrate hosts, and phlebotomine sand fly vectors represent a public health challenge in Latin America. This study aimed to investigate the serological and molecular occurrence of *Leishmania* spp. in dogs and cats from São Luís, Maranhão, northeastern Brazil.

**Methods:**

Blood samples from 330 dogs and 21 cats were analyzed by indirect immunofluorescence antibody test (IFAT) using *Leishmania infantum* and *Leishmania amazonensis* antigens and by ITS1 Polymerase Chain Reaction–Restriction Fragment Length Polymorphism (ITS1 PCR–RFLP).

**Results:**

In dogs, seroreactivity against *L. amazonensis* and *L. infantum* antigens was detected in 37.9% and 23.0%, respectively, and *Leishmania* DNA in 58/330 (17.6%). Of nine canine samples successfully characterized by PCR–RFLP, eight showed profiles compatible with *L. infantum* and one a mixed profile suggestive of both species. In cats, seroreactivity against *L. amazonensis* and *L. infantum* antigens was detected in 28.6% and 14.3%, respectively, and *L. infantum* DNA was identified in two animals. Univariable analysis showed associations between seroreactivity to *L. infantum* antigen and age, sex, and clinical signs, whereas seroreactivity to *L. amazonensis* antigen was associated with age, sex, street access, and the reported presence of wild animals around the household.

**Conclusion:**

The higher seroreactivity to *L. amazonensis* antigen should be interpreted cautiously because of potential serological cross-reactivity and limited species-level molecular characterization. Together, the findings raise the possibility that *Leishmania* occurrence in domestic animals in this endemic area may not be restricted to *L. infantum*, warranting further species-specific investigation.

## Introduction

Leishmaniasis is a complex vector-borne disease caused by protozoa of the genus *Leishmania*, transmitted by phlebotomine sandflies, and remains a major public health concern in tropical and subtropical regions, affecting both humans and domestic animals. In the Americas, visceral leishmaniasis (VL) is primarily associated with *Leishmania infantum* [8, 11]. American tegumentary leishmaniasis is associated with several *Leishmania* species, with *Leishmania (Viannia) braziliensis* recognized as the predominant species in the Americas and particularly in Brazil, while *Leishmania (Leishmania) amazonensis* and *Leishmania (Viannia) guyanensis* are also important etiological agents [16, 44].

These transmission cycles involve interactions among *Leishmania* species, vertebrate hosts, and diverse phlebotomine sand fly vectors, whose ecological and geographic distributions contribute to distinct regional transmission patterns [34, 38]. In anthropized environments, domestic and peridomestic transmission may overlap with sylvatic cycles, as vertebrate hosts occupying different ecological habitats can contribute to connections between these transmission interfaces [2, 38].

However, the classical clinical–epidemiological distinction is being challenged by evidence of overlapping transmission cycles in peri-urban and anthropized environments. Studies suggest that ecological overlap, host permissiveness, and environmental changes may allow different species to circulate together, complicating the traditional dichotomous classification [25, 50].

In Brazil, visceral leishmaniasis has undergone a marked epidemiological transition over recent decades, shifting from predominantly rural transmission to sustained urban and peri-urban cycles. This expansion reflects the adaptability of *Leishmania* spp. and their vectors to human-modified environments, facilitating closer contact between vectors, domestic reservoirs, and human populations. Environmental degradation, migration of human and animal populations, and precarious housing are recognized as important factors contributing to parasite persistence and geographic expansion [26, 50].

Climate change may further influence leishmaniasis transmission by altering environmental conditions that affect vector distribution and disease occurrence, with potential changes in the geographic distribution of leishmaniasis in Brazil [23]. Despite the implementation of national surveillance and control strategies, transmission remains intense in several urban centers, particularly in northeastern Brazil, where endemicity persists under complex socio-environmental conditions [26, 50].

Domestic dogs are considered the main urban reservoir of *L. infantum*; however, growing attention has been given to other domestic species, particularly cats, whose epidemiological role remains incompletely understood [12]. *L. infantum* and *Leishmania braziliensis* are considered the most epidemiologically relevant species in canine leishmaniasis in South America. Notably, *L. amazonensis* has been isolated from dogs with visceral manifestations, highlighting atypical host–parasite interactions and the need for species-level identification. These findings reinforce the need for species-level molecular discrimination in endemic settings, as distinct *Leishmania* species may coexist in the same area [14]. Importantly, the occurrence of multiple *Leishmania* species is not restricted to domestic animals, as coinfection has also been documented in small mammals in northeastern Brazil, including the simultaneous detection of *L. (Viannia)* spp. and *L. (L.) infantum* in different host species [19].

Beyond visceral leishmaniasis, the increasing domestic transmission of American Tegumentary Leishmaniasis (ATL) has also raised concerns regarding the epidemiological role of dogs [18]. Likewise, there is no consistent evidence supporting the role of cats in the epidemiological chain of the disease, although infected animals are frequently detected in endemic areas [13]. These uncertainties highlight gaps in understanding host participation and transmission dynamics, particularly where multiple *Leishmania* species may coexist. Accurate diagnosis and species identification are essential, especially in endemic regions, in order to characterize the epidemiological profile of the disease and support effective control strategies [39, 43].

Reports of molecular detection of atypical species in domestic animals have raised questions regarding under-recognized transmission patterns in endemic areas. However, the frequency, distribution, and epidemiological significance of co-circulating *Leishmania* species in domestic environments remain poorly understood, particularly in urban endemic settings [33].

In this context, São Luís, in northeastern Brazil, represents a relevant epidemiological setting, characterized by sustained visceral leishmaniasis transmission, peri-urban expansion, and environmental conditions that may favor the interaction between vectors, domestic animals, and wildlife. Phlebotomine fauna reported in the metropolitan region includes *Lutzomyia longipalpis* and *Lutzomyia whitmani* among the most abundant species, as well as *Lutzomyia flaviscutellata*, vectors associated with visceral and tegumentary leishmaniasis transmission cycles [30].

Therefore, this study aimed to investigate the serological and molecular occurrence of *Leishmania* spp. in domestic dogs and cats from an endemic area for visceral leishmaniasis in northeastern Brazil, with special emphasis on identifying potential co-circulation of *L. infantum* and *L. amazonensis*. This approach was intended to explore whether the epidemiological context of *Leishmania* infection in domestic animals in this area could extend beyond the classical urban cycle predominantly associated with *L. infantum*.

## Methods

### Ethical Approval

This study was approved by the Ethics Committee on Animal Use of the State University of Maranhão (CEEA/UEMA no. 09/2018) and by the Brazilian National Research Ethics Commission (Plataforma Brasil). Written informed consent was obtained from all animal owners prior to sample collection.

### Study Area

**Fig. 1:**
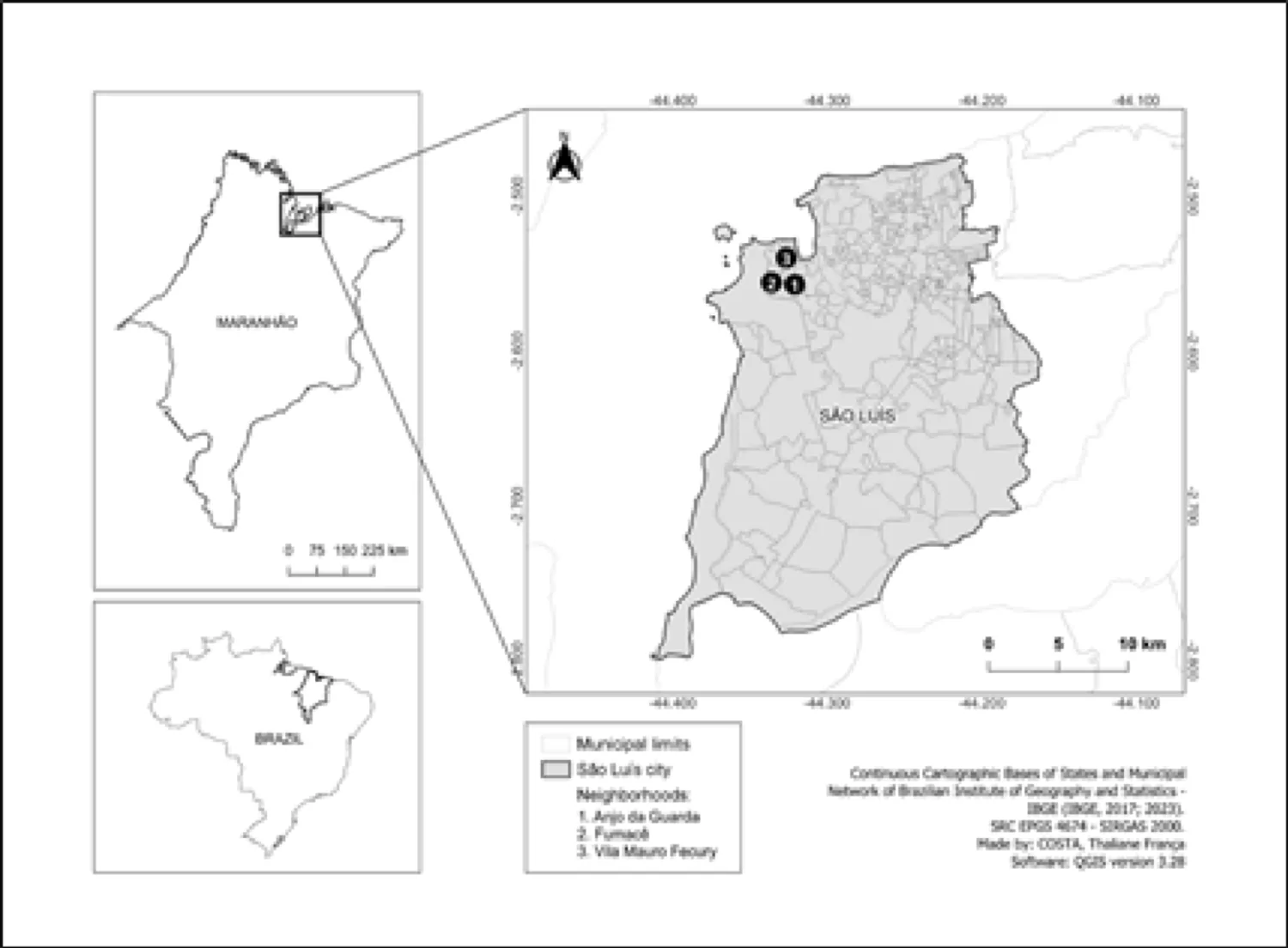
Maranhão State map, highlighting the city of São Luís and the three studied locations

The study was conducted in three peri-urban neighborhoods of São Luís (2° 31′ S, 44° 18′ W), Maranhão State, northeastern Brazil — Fumacê (2° 33′ 36.4″ S, 44° 20′ 23.9″ W), Anjo da Guarda (2° 33′ 45.1″ S, 44° 19′ 35.0″ W), and Mauro Fecury (2° 32′ 32.5″ S, 44° 19′ 54.2″ W) (Fig. 1). These neighborhoods are located in the Itaqui-Bacanga region, which comprises residential neighborhoods and major port and industrial activities [1]. The region also presents limitations in urban infrastructure and sanitation, including restricted sewage coverage [40]. The municipality has a tropical humid climate with marked seasonal rainfall and is historically endemic for visceral leishmaniasis [42]. According to the Brazilian Notifiable Diseases Information System (SINAN), 205 confirmed human cases of visceral leishmaniasis were reported among residents of São Luís between 2020 and 2025, indicating continued transmission in the municipality [7].

### Study Design and Sample Size

A cross-sectional study was conducted using convenience sampling of domiciled dogs and cats older than six months.

Sample size for dogs was estimated using the StatCalc module of Epi Info (CDC), considering a finite population. In the absence of previous prevalence data for the canine population in the study area, an expected prevalence of 50% was adopted as a conservative assumption for sample size estimation [49], with a 95% confidence level and 5% precision, resulting in an adjusted sample size of 323 dogs, which was rounded up to 330 animals. The estimated canine population in the study area was approximately 2,035 animals, based on a ratio of one dog per 10 human inhabitants, as previously used in a Brazilian epidemiological study of canine visceral leishmaniasis [3], and a human population of approximately 20,355 inhabitants in the sampled area, according to data provided by the Municipal Health Department.

No formal sample size calculation was performed for cats. Feline sampling (n = 21) was exploratory and resulted in a smaller sample because cats were less frequently available than dogs in the households visited. In addition, feline sampling was constrained by difficulties in restraint and blood collection, and some owners declined blood collection because of concerns about handling-related stress.

### Sample Collection

Sampling was conducted from July to October 2020 in the three urban neighborhoods included in the study (Fumacê, Anjo da Guarda, and Mauro Fecury). Households were recruited door-to-door with the assistance of local Community Health Agents. All eligible domiciled dogs and cats older than six months whose owners agreed to participate were included, with no predefined limit on the number of animals sampled per household. Thus, when multiple eligible animals were present in a participating household, all could be sampled. A total of 110 dogs were sampled in each neighborhood, resulting in 330 dogs being included in the study.

Peripheral blood was collected by cephalic or jugular venipuncture. Serum was separated by centrifugation and stored at −20 °C until serological testing. Whole blood aliquots were preserved in ethanol at a 1:1 (v/v) blood-to-ethanol ratio immediately after collection in the field, following Pacheco et al. [29]. The samples were transported under refrigeration in insulated containers with reusable ice packs until arrival at the laboratory and subsequently stored at −20 °C for molecular analyses. Peripheral blood was selected due to its minimally invasive nature, which ensures higher owner compliance in large-scale epidemiological surveys, allowing for a more representative population sampling.

### Epidemiological Data

Owners completed a structured questionnaire addressing animal characteristics (age, sex, clinical signs, lifestyle), environmental conditions (presence of organic matter, other animals, previous leishmaniasis cases), and preventive measures. Specifically, the epidemiological variables included age, sex, access to the street, mode of acquisition of the animal, accumulation of organic matter, presence of other animals in the household, presence of another animal with suspected or diagnosed leishmaniasis in the household, history of *Leishmania*-positive animals or human cases in the household, presence of wild animals around the household, peridomiciliary characteristics, and adoption of preventive and control measures.

The presence of wild animals around the household was recorded based on information reported by the owners. These animals were not necessarily classified as synanthropic. Access to the street was defined as whether the animal was allowed to leave the household premises. Preventive and control measures included the use of insecticide-impregnated collars for dogs, environmental management and removal of organic matter from the peridomicile, insecticide use, installation of screens on doors and windows, use of repellents or mosquito nets, and routine veterinary monitoring of companion animals. Clinical evaluation included the recording of signs compatible with canine leishmaniasis, including cutaneous ulcers, diffuse alopecia, dermatitis, periocular scaling, onychogryphosis, lymphadenomegaly, hepatosplenomegaly, mucosal pallor, keratoconjunctivitis sicca, cachexia and other clinical abnormalities observed during physical examinations. These clinical findings were recorded as epidemiological variables and were not considered diagnostic confirmation of canine leishmaniasis.

### Serological Analysis

Indirect immunofluorescence antibody tests (IFAT) were performed for *L. infantum* and *L. amazonensis* as previously described [27]. Promastigote forms cultured in vitro were used as antigens. Serum samples were screened at a cutoff dilution of 1:80, as previously adopted for IFAT-based serological detection of anti-*Leishmania* antibodies in dogs and cats [28, 32]. The same cutoff was applied to both *L. infantum* and *L. amazonensis* antigens to standardize the serological comparison between the two antigens. IFAT was selected in accordance with the Brazilian Ministry of Health guidelines available at the time the study was conducted [6]. Positive samples were serially diluted two-fold to determine endpoint titers. Samples were considered positive when clear peripheral fluorescence of promastigotes was observed.

### DNA Extraction and PCR Amplification

Genomic DNA was extracted using the PureLink™ Genomic DNA Mini Kit (Thermo Fisher Scientific) following the manufacturer’s instructions. The ITS1 region was amplified using primers described by Schönian et al. [41]. PCR reactions were performed in a final volume of 20 µL containing 12.7 µL of ultrapure water, 2 µL of 10× PCR buffer, 0.8 µL of MgCl₂ (50 mM), 2 µL of dNTPs (2 mM), 0.1 µL of Taq DNA polymerase (5 U/µL; Invitrogen®, Carlsbad, USA), 0.2 µL of each primer (10 µM), and 2 µL of template DNA. The thermal cycling conditions consisted of an initial denaturation at 95 °C for 3 min, followed by 35 cycles of denaturation at 95 °C for 30 s, annealing at 53 °C for 30 s, and extension at 72 °C for 1 min, with a final extension at 72 °C for 5 min. Reference DNA from *L. infantum* and *L. amazonensis*, obtained from the Brazilian Trypanosomatid Collection, was included as positive-control material, whereas ultrapure water was used as the negative control to monitor contamination. Samples showing an amplification product of approximately 300–350 bp, corresponding to the expected ITS1 fragment, were considered PCR-positive**.** Amplified products were visualized on 1.5% agarose gels stained with SYBR Safe.

### Digestion of Polymerase Chain Reaction Fragments

To differentiate *Leishmania* species, all PCR-positive products were subjected to restriction digestion with HaeIII according to Schönian et al. [41]. Each digestion reaction contained 12.5 µL of purified PCR product, 2 µL of 10× reaction buffer, 1 µL of HaeIII restriction enzyme, and ultrapure water to complete the final reaction volume. PCR amplification and restriction digestion were repeated when necessary. Only restriction profiles that were sufficiently clear and interpretable by comparison with the reference controls were used for species characterization. Samples yielding absent, weak, or poorly defined restriction profiles were not assigned to a *Leishmania* species.

The restriction profiles obtained from the samples were compared with those of *L. infantum* and *L. amazonensis* reference controls. Digested fragments were separated on 2% agarose gels and visualized under UV light to determine species-specific banding patterns.

### Statistical Analysis

Data were analyzed using EpiInfo version 6.0 (CDC, Atlanta, USA). Associations between categorical variables were evaluated using Chi-square or Fisher’s exact test, as appropriate. Crude odds ratios (ORs) and their respective 95% confidence intervals (95% CIs) were calculated to quantify the magnitude and precision of the associations identified in the univariable analysis. The epidemiological analyses were considered exploratory, and no multivariable adjustment was performed. Statistical significance was set at *p* < 0.05.

## Results

### Study Population

A total of 330 domiciled dogs and 21 cats were included in this study. Among dogs, 165 (50%) were male and 165 (50%) were female, and the majority were older than one year (254/330; 76.96%). Among cats, 11 (52.38%) were male, and most were older than one year (16/21; 76.19%).

### Serological Findings in Dogs and Cats

**Fig. 2:**
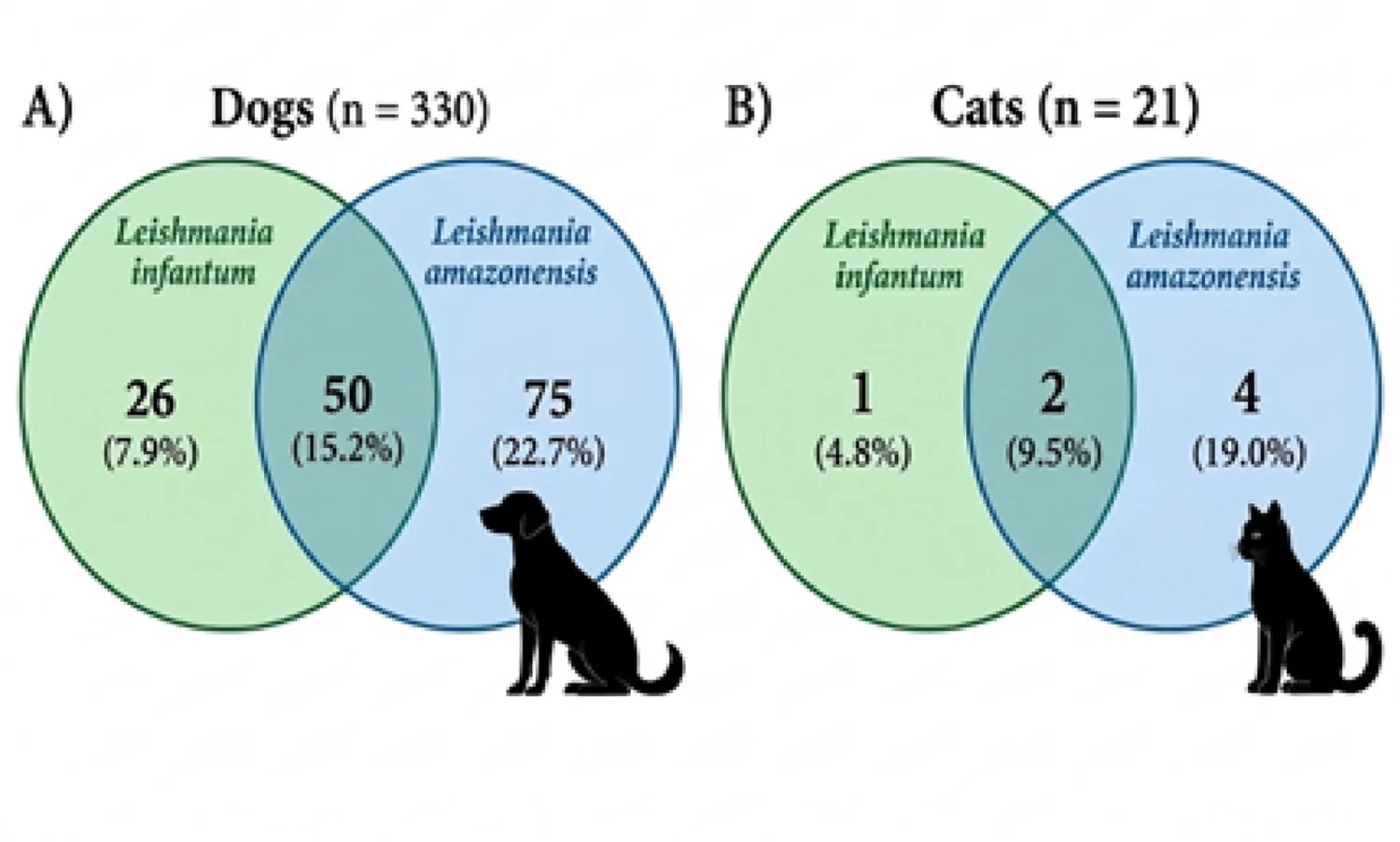
Venn diagrams showing seroreactivity to *Leishmania infantum* and *Leishmania amazonensis* antigens in dogs (**A**) and cats (**B**)

Seroreactivity against *L. amazonensis* antigen was detected in 125/330 (37.9%) dogs and against *L. infantum* antigen in 76/330 (23.0%). These totals include animals seroreactive to both antigens. Accordingly, among the 330 dogs, 75 (22.7%) were seroreactive only to *L. amazonensis* antigen, 26 (7.9%) only to *L. infantum* antigen, and 50 (15.2%) to both antigens (Fig. 2A). Antibody titers among the 125 dogs seroreactive to *L. amazonensis* antigen and the 76 dogs seroreactive to *L. infantum* antigen ranged from 1:80 to 1:5,120 (Table 1). Among dogs seroreactive against *L. amazonensis* antigen, 74/125 (59.2%) were male and 107/125 (85.6%) were older than one year.

**Table 1:** Anti-*Leishmania infantum* and *Leishmania amazonensis* antibody titers in dogs and cats tested by indirect immunofluorescence antibody test (IFAT)

	*Leishmania infantum*		*Leishmania amazonensis*	
Titer	Dogs n (%)	Cats n (%)	Dogs n (%)	Cats n (%)
1:80	29 (38.16)	3 (100.00)	40 (32.00)	4 (66.67)
1:160	23 (30.26)	–	38 (30.40)	2 (33.33)
1:320	15 (19.74)	–	31 (24.80)	–
1:640	7 (9.21)	–	8 (6.40)	–
1:1,280	2 (2.63)	–	6 (4.80)	–
1:2,560	–	–	1 (0.80)	–
1:5,120	–	–	1 (0.80)	–
Total	76 (100.00)	3 (100.00)	125 (100.00)	6 (100.00)

Clinical signs compatible with canine leishmaniasis were recorded in 16/76 (21.1%) dogs seroreactive to *L. infantum* antigen, including cutaneous ulcers, diffuse alopecia, dermatitis, onychogryphosis, cachexia, hepatosplenomegaly and lymphadenomegaly.

In cats, seroreactivity against *L. amazonensis* antigen was detected in 6/21 (28.6%) animals and against *L. infantum* antigen in 3/21 (14.3%). These totals include animals seroreactive to both antigens. Accordingly, among the 21 cats, 4 (19.0%) were seroreactive only to *L. amazonensis* antigen, 1 (4.8%) only to *L. infantum* antigen, and 2 (9.5%) to both antigens (Fig. 2B). Antibody titers among the six cats seroreactive to *L. amazonensis* antigen and the three cats seroreactive to *L. infantum* antigen ranged from 1:80 to 1:160 (Table 1). One seroreactive cat showed cutaneous lesions.

### Molecular Detection and Species Identification

PCR detected *Leishmania* DNA in 58/330 (17.6%) dogs and 2/21 (9.5%) cats. Molecular identification via PCR–RFLP targeting the ITS1 region allowed for the differentiation of *Leishmania* species based on well-established restriction profiles. Species identification was achieved in 9/58 PCR-positive canine samples. The remaining 49 PCR-positive canine samples did not yield restriction profiles of sufficient quality for reliable species assignment and therefore remained uncharacterized at the species level. Eight samples showed restriction fragments characteristic of *L. infantum*, while one sample demonstrated a mixed restriction pattern, suggesting the presence of genomic fragments from both *L. infantum* and *L. amazonensis*.

Among the eight canine samples showing an *L. infantum* restriction profile, four were seroreactive only to *L. infantum* antigen and four were seroreactive to both *L. infantum* and *L. amazonensis* antigens. The canine sample showing the mixed restriction profile was also seroreactive to both antigens. Both PCR-positive feline samples showed patterns compatible with *L. infantum*. Both cats were seronegative by IFAT for *L. infantum* and *L. amazonensis*.

### Integrated Analysis of Serological and Molecular Findings and Associated Factors

Among PCR-positive dogs (n = 58), 14 (24.14%) were also seroreactive to *L. infantum* antigen, 20 (34.48%) to *L. amazonensis* antigen, including eight dogs that were seroreactive to both antigens, and 32 (55.17%) were seronegative by IFAT for both antigens.

For *L. infantum*, univariable analysis showed significant associations between seroreactivity and age greater than one year (OR = 2.27; 95% CI 1.10–4.67; *p* = 0.023) (Table 2), male sex (OR = 1.81; 95% CI 1.07–3.07; *p* = 0.025), and the presence of clinical signs compatible with canine leishmaniasis (OR = 2.87; 95% CI 1.42–5.81; *p* = 0.002).

**Table 2:** Univariable analysis of factors associated with seroreactivity to *Leishmania infantum* antigen in dogs from São Luís, Maranhão, Brazil

Variable	Category	Positive/Exposed	%	Negative/Exposed	%	Crude OR (95% CI)	*P* value
Access to the street	No	15/56	26.79	41/56	73.21	1.00	0.426
	Yes	60/274	21.90	214/274	78.1	0.77 (0.40–1.48)	
Accumulation of organic matter	No	9/35	25.71	26/35	74.29	1.00	0.656
	Yes	66/295	22.37	229/295	77.63	0.83 (0.37–1.86)	
Animal with suspected or diagnosed leishmaniasis in the household	No	66/285	23.16	219/285	76.84	1.00	0.639
	Yes	9/45	20.00	36/45	80	0.83 (0.38–1.81)	
Other animals in the household	No	38/147	25.85	109/147	74.15	1.00	0.225
	Yes	37/183	20.22	146/183	79.78	0.73 (0.43–1.22)	
History of an animal leishmaniasis case	No	69/309	22.33	240/309	77.67	1.00	0.509
	Yes	6/21	28.57	15/21	71.43	1.39 (0.52–3.72)	
History of a human leishmaniasis case	No	75/329	22.80	254/329	77.2	1.00	1.000†
	Yes	0/1	0.00	1/1	100	NE	
Adoption of control measures	No	38/185	20.54	147/185	79.46	1.00	0.284
	Yes	37/145	25.52	108/145	74.48	1.33 (0.79–2.22)	
Age	< 1 year	10/76	13.16	66/76	86.84	1.00	0.023*
	>1 year	65/254	25.59	189/254	74.41	2.27 (1.10–4.67)	
Reported presence of wild animals around the household	No	10/70	14.29	60/70	85.71	1.00	0.058
	Yes	65/260	25.00	195/260	75	2.00 (0.97–4.13)	
Sex	Female	29/165	17.58	136/165	82.42	1.00	0.026*
	Male	46/165	27.88	119/165	72.12	1.81 (1.07–3.07)	
Presence of clinical signs compatible with canine leishmaniasis	No	59/292	20.21	233/292	79.79	1.00	0.002*
	Yes	16/38	42.11	22/38	57.89	2.87 (1.42–5.81)	
Peridomicile	Residences	44/197	22.34	153/197	77.66	1.00	0.836
	Wasteland	31/133	23.31	102/133	76.69	1.06 (0.63–1.78)	
How acquired the animal?	Bought	9/44	20.45	35/44	79.55	1.00	0.699
	Adopted	66/286	23.08	220/286	76.92	1.17 (0.53–2.55)	

*Statistically significant (p < 0.05)

^†^Fisher's exact test

*OR* Odds ratio; *CI* Confidence interval; *NE* Not estimable. The first category of each variable was used as the reference category (OR = 1.00)

For *L. amazonensis*, significant associations were observed with age greater than one year (*p* = 0.003), male sex (*p* = 0.009), street access (*p* = 0.002), and presence of wild animals around the household (*p* = 0.003) (Table 3).

**Table 3:** Univariable analysis of factors associated with seroreactivity to *Leishmania amazonensis* antigen in dogs from São Luís, Maranhão, Brazil

Variable	Category	Positive/Exposed	%	Negative/Exposed	%	Crude OR (95% CI)	*P* value
Access to the street	No	11/56	19.64	45/56	80.36	1.00	0.002*
	Yes	114/274	41.61	160/274	58.39	2.91 (1.45–5.88)	
Accumulation of organic matter	No	13/35	37.14	22/35	62.86	1.00	0.924
	Yes	112/295	37.97	183/295	62.03	1.04 (0.50–2.14)	
Animal with suspected or diagnosed leishmaniasis in the household	No	110/285	38.60	175/285	61.40	1.00	0.498
	Yes	15/45	33.33	30/45	66.67	0.80 (0.41–1.55)	
Other animals in the household	No	58/147	39.46	89/147	60.54	1.00	0.596
	Yes	67/183	36.61	116/183	63.39	0.89 (0.57–1.39)	
History of an animal leishmaniasis case	No	118/309	38.19	191/309	61.81	1.00	0.657
	Yes	7/21	33.33	14/21	66.67	0.81 (0.32–2.06)	
History of a human leishmaniasis case	No	125/329	37.99	204/329	62.01	1.00	1.000†
	Yes	0/1	0.00	1/1	100.00	NE	
Adoption of control measures	No	70/185	37.84	115/185	62.16	1.00	0.986
	Yes	55/145	37.93	90/145	62.07	1.00 (0.64–1.57)	
Age	< 1 year	18/76	23.68	58/76	76.32	1.00	0.003*
	>1 year	107/254	42.13	147/254	57.87	2.35 (1.31–4.21)	
Reported presence of wild animals around the household	No	16/70	22.86	54/70	77.14	1.00	0.003*
	Yes	109/260	41.92	151/260	58.08	2.44 (1.32–4.48)	
Sex	Female	51/165	30.91	114/165	69.09	1.00	0.009*
	Male	74/165	44.85	91/165	55.15	1.82 (1.16–2.85)	
Presence of clinical signs compatible with canine leishmaniasis	No	106/292	36.30	186/292	63.70	1.00	0.101
	Yes	19/38	50.00	19/38	50.00	1.75 (0.89–3.46)	
Peridomicile	Residences	68/197	34.52	129/197	65.48	1.00	0.125
	Wasteland	57/133	42.86	76/133	57.14	1.42 (0.91–2.24)	
How acquired the animal?	Bought	20/44	45.45	24/44	54.55	1.00	0.265
	Adopted	105/286	36.71	181/286	63.29	0.70 (0.37–1.32)	

*Statistically significant (*p* < 0.05)

^†^Fisher's exact test

*OR* Odds ratio; *CI* Confidence interval; *NE* Not estimable

## Discussion

This study investigated the occurrence of *Leishmania* spp. in domestic dogs and cats from a visceral leishmaniasis–endemic area in northeastern Brazil, focusing on the potential co-circulation of *L. infantum* and *L. amazonensis*. In dogs, seroreactivity to both *L. infantum* and *L. amazonensis* antigens were detected, while molecular characterization predominantly identified *L. infantum*, with one sample showing a mixed restriction profile suggestive of the presence of both species.

Serological analysis showed greater reactivity to *L. amazonensis* than to *L. infantum* in dogs. Previous studies in Brazil have reported variable seroprevalence rates for *L. amazonensis*, ranging from 3.5 to 74.8%, depending on the region and diagnostic method employed [20, 39]. In the present study, the higher seroreactivity to *L. amazonensis* antigen is noteworthy in an area traditionally recognized as endemic for visceral leishmaniasis, although this finding alone cannot be interpreted as evidence of species-specific infection.

The occurrence of distinct serological profiles, including animals with higher titers for *L. amazonensis* than for *L. infantum*, is noteworthy. However, serological reactivity alone does not necessarily indicate active or species-specific infection [37], particularly in areas where different *Leishmania* species may co-exist and serological cross-reactivity may occur.

Molecular detection identified *Leishmania* DNA in 58/330 (17.6%) canine samples, although interpretable PCR–RFLP profiles were obtained for only 9/58 PCR-positive samples. The remaining samples yielded absent, weak, or poorly defined restriction profiles and were therefore not assigned to a species. Although the use of peripheral blood may pose challenges for molecular detection because of low parasite loads [17, 51], it cannot alone explain the limited PCR–RFLP characterization observed here. Among the successfully characterized samples, *L. infantum* predominated, while one mixed restriction profile suggested the presence of both *L. infantum* and *L. amazonensis*. This finding is noteworthy in the context of seroreactivity to both antigens but should be interpreted cautiously given the limited number of samples characterized at the species level.

The discrepancy between serological and molecular results, including PCR-positive/seronegative animals, may reflect differences in infection stage, immune response, or sample type [15]. Similar discordant findings have been reported in endemic regions [26]. The limited molecular characterization, despite the higher seroreactivity to *L. amazonensis* antigen, may reflect differences in parasite load, sample type, or other methodological factors and should therefore be interpreted cautiously.

Factors associated with seroreactivity to *L. infantum* antigen included age greater than one year, male sex, and clinical signs, whereas seroreactivity to *L. amazonensis* antigen was associated with age greater than one year, male sex, street access, and the reported presence of wild animals around the household. These findings are consistent with previous reports suggesting that greater environmental exposure may be associated with increased contact with infected sand flies [5]. Adult animals may accumulate vector exposure over time, and roaming behavior may increase opportunities for vector contact.

Clinical signs compatible with canine leishmaniasis were observed in a subset of seroreactive dogs, consistent with the variable clinical presentation reported for canine leishmaniasis. Previous research has shown that infection does not necessarily lead to clinical symptoms, and a significant proportion of infected dogs remain asymptomatic [4, 45]. The wide range of clinical manifestations, extending from asymptomatic infections to severe disease, is influenced by the host's immune response and parasite load [46]. When observed in this study, these clinical signs were among the most common reported in canine leishmaniasis.

In cats, seroreactivity was lower than in dogs, and molecular detection was limited to two samples identified as *L. infantum*. Reports of feline infection by *L. amazonensis* remain rare in Brazil [9, 47, 48]. The molecular detection of *L. infantum* in two cats confirms the occurrence of feline infection in the study area, consistent with previous reports indicating that infection in cats is generally subclinical or associated with dermatological symptoms [31]. The small feline sample size in this study limits any inference regarding their epidemiological role, but seroreactivity and molecular detection were observed, and their contribution to transmission dynamics remains a subject for further investigation.

Environmental characteristics reported in the study areas—including limitations in sanitation and sewage infrastructure and the reported presence of animals such as *Mus musculus* and *Didelphis marsupialis* around households—may favor vector maintenance [22]. Although entomological surveys were not performed, previous reports have documented the presence of competent sandfly vectors in São Luís [21, 35], supporting the plausibility of active transmission in these peri-urban settings.

The vector component is also relevant when considering the possible occurrence of different *Leishmania* species in this setting. *Lutzomyia longipalpis*, the principal vector of *L. infantum* in the Americas, is considered a permissive sand fly vector because it can support the development of different *Leishmania* species, whereas selective vectors exhibit more restricted parasite–vector compatibility [36, 52]. Although *L. amazonensis* is classically associated with *Lutzomyia flaviscutellata*, its DNA has also been detected in naturally infected *Lu. longipalpis* in northeastern Brazil [10]. These observations illustrate that vector–parasite associations may be more complex than a strict correspondence between a single sand fly and a single *Leishmania* species. However, permissiveness under experimental conditions or detection of parasite DNA in field-collected sand flies does not establish their role in natural transmission. Because no entomological survey was performed in the present study, the serological and molecular findings in domestic animals cannot be attributed to any particular vector species.

The animal findings observed in this study should also be considered within the local epidemiological context of human visceral leishmaniasis. The Itaqui-Bacanga region was involved in the urban expansion of visceral leishmaniasis in São Luís, and Anjo da Guarda, one of the neighborhoods investigated here, was among the areas affected by human cases during the urbanization and geographic expansion of the disease [24]. More recent surveillance data indicate that human VL continues to occur in the municipality, with 205 confirmed cases reported between 2020 and 2025 [7]. In this context, the detection of *Leishmania* DNA in dogs and the predominance of *L. infantum* among the successfully characterized samples are consistent with the endemic context of VL in São Luís. However, because human infection was not investigated and the available surveillance data refer to the municipality as a whole, no direct relationship between the animal findings and the occurrence of human cases in the three neighborhoods can be established.

Importantly, seroreactivity to *L. amazonensis* antigen, together with the detection of a mixed PCR–RFLP profile in one dog, suggests the possibility of involvement of different *Leishmania* species in this epidemiological setting, although the mixed profile alone does not confirm coinfection.

This study has limitations, including the exclusive use of peripheral blood for molecular detection, the low number of feline samples, and the reduced success rate of species identification by PCR–RFLP. Additionally, the absence of tissue sampling and entomological investigation may have limited the detection of parasite diversity and transmission dynamics in the study area.

Because the epidemiological associations were based on univariable analyses, potential confounding among the evaluated factors cannot be excluded. Therefore, these findings should be interpreted as exploratory associations rather than independent risk factors.

In conclusion, seroreactivity to both *L. infantum* and *L. amazonensis* antigens was detected in domestic dogs and cats from a visceral leishmaniasis–endemic area, while molecular characterization predominantly identified *L. infantum*. The higher seroreactivity to *L. amazonensis* antigen in dogs, together with the mixed PCR–RFLP profile observed in one animal, raises the possibility that *Leishmania* occurrence in domestic animals in this area may not be restricted to *L. infantum*. However, these findings should be interpreted cautiously considering potential serological cross-reactivity and the limited species-level molecular characterization. Overall, the results highlight the value of considering different *Leishmania* species in epidemiological investigations and of combining serological and molecular approaches to better understand *Leishmania* occurrence in urban endemic settings.

## Data Availability

The datasets generated during and/or analyzed during the current study are available from the corresponding author on reasonable request.

## References

[1] Andrade MMN, Szlafsztein CF, Souza-Filho PWM, Araújo AR, Gomes MKT (2010) A socioeconomic and natural vulnerability index for oil spills in an Amazonian harbor: A case study using GIS and remote sensing. J Environ Manage 91:1972–1980. 10.1016/j.jenvman.2010.04.016 20627540

[2] Andrade MS, Courtenay O, Brito MEF, Carvalho FG, Carvalho AWS, Soares F, Carvalho SM, Costa PL, Zampieri R, Floeter-Winter LM, Shaw JJ, Brandão-Filho SP (2015) Infectiousness of sylvatic and synanthropic small rodents implicates a multi-host reservoir of *Leishmania (Viannia) braziliensis*. PLoS Negl Trop Dis 9:e0004137. 10.1371/journal.pntd.0004137 PMC4598029 26448187

[3] Azevedo MAA, Dias AKK, Paula HB, Perri SHV, Nunes CM (2008) Avaliação da leishmaniose visceral canina em Poxoréo, Estado do Mato Grosso. Brasil Rev Bras Parasitol Vet 17:123–127. 10.1590/S1984-29612008000300001 19245756

[4] Baneth G, Koutinas AF, Solano-Gallego L, Bourdeau P, Ferrer L (2008) Canine leishmaniosis – new concepts and insights on an expanding zoonosis: part one. Trends Parasitol 24:324–330. 10.1016/j.pt.2008.04.001 18514028

[5] Barbosa, D.S., Rocha, A.L., Santana, A.A., Souza, C. da S.F., Dias, R.A., Costa-Junior, L.M., Abreu-Silva, A.L., 2010. Soroprevalência e variáveis epidemiológicas associadas à leishmaniose visceral canina em área endêmica no município de São Luís, Maranhão, Brasil. Ci. Anim. Bras. 11, 653–659. 10.5216/cab.v11i3.5933

[6] Brazil. Ministério da Saúde. Secretaria de Vigilância em Saúde. Departamento de Vigilância Epidemiológica (2014) Manual de vigilância e controle da leishmaniose visceral, 1st ed., 5th reprint. Ministério da Saúde, Brasília, DF

[7] Brazilian Ministry of Health, 2026. Notifiable Diseases Information System (SINAN): Visceral leishmaniasis – confirmed cases notified in the Notifiable Diseases Information System, São Luís, Maranhão, 2020–2025. DATASUS, Ministry of Health, Brazil. Accessed 12 September 2026

[8] Burza S, Croft SL, Boelaert M (2018) Leishmaniasis. Lancet 392:951–970. 10.1016/S0140-6736(18)31204-2 30126638

[9] Carneiro LA, Vasconcelos Dos Santos T, Lima LVDR, Ramos PKS, Campos MB, Silveira FT (2020) First report on feline leishmaniasis caused by *Leishmania (Leishmania) amazonensis* in Amazonian Brazil. Vet Parasitol Reg Stud Reports 19:100360. 10.1016/j.vprsr.2019.100360 32057387

[10] Carvalho-Silva R, Ribeiro-da-Silva RC, Cruz LNPD, Oliveira MS, Amoedo PM, Rebêlo JMM, Guimarães-e-Silva AS, Pinheiro VCS (2022) Predominance of *Leishmania (Leishmania) amazonensis* DNA in *Lutzomyia longipalpis* sand flies (Diptera: Psychodidae) from an endemic area for leishmaniasis in Northeastern Brazil. Rev Inst Med Trop São Paulo 64:e32. 10.1590/S1678-9946202264032 PMC9084467 35544910

[11] Costa, A.P., Costa, F.B., Soares, H.S., Ramirez, D.G., Araújo, A. de C., Ferreira, J.I.G. da S., Tonhosolo, R., Dias, R.A., Gennari, S.M., Marcili, A., 2015. Environmental factors and ecosystems associated with canine visceral leishmaniasis in Northeastern Brazil. Vector Borne Zoonotic Dis. 15, 765–774. 10.1089/vbz.2015.1866 26684524

[12] Dantas-Torres F, Miró G, Baneth G, Bourdeau P, Breitschwerdt E, Capelli G, Cardoso L, Day MJ, Dobler G, Ferrer L, Irwin P, Jongejan F, Kempf VAJ, Kohn B, Lappin M, Little S, Madder M, Maggi R, Maia C, Marcondes M, Naucke T, Oliva G, Pennisi MG, Penzhorn BL, Peregrine A, Pfeffer M, Roura X, Sainz A, Shin S, Solano-Gallego L, Straubinger RK, Tasker S, Traub R, Wright I, Bowman DD, Gradoni L, Otranto D (2019) Canine leishmaniasis control in the context of One Health. Emerg Infect Dis 25:1–4. 10.3201/eid2512.190164 PMC6874277 31742505

[13] De Sousa Lima, T.S.N., Gondim, N.M., Guimarães, L., Cavalcante, L.A., Siebra, C.C., de Lima, M.S., Mota Filho, A.C., André, W.P.P., 2024. Clinical, diagnostic and therapeutic aspects of feline leishmaniasis: literature review. Braz. J. Anim. Environ. Res. 7, 99–112. 10.34188/bjaerv7n1-009

[14] Diotallevi A, Buffi G, Ceccarelli M, Neitzke-Abreu HC, Gnutzmann LV, Da Costa Lima MS, Galluzzi L (2020) Real-time PCR to differentiate among *Leishmania (Viannia)* subgenus, *Leishmania (Leishmania) infantum* and *Leishmania (Leishmania) amazonensis*: application on Brazilian clinical samples. Acta Trop 201:105178. 10.1016/j.actatropica.2019.105178 31606374

[15] Evaristo, A.M.D.C.F., Araujo, A.C., da Costa, A.P., Sales, K.G.D.S., da Silva, J.A.M., Dantas-Torres, F., Horta, M.C., 2021. Comparison of serological and molecular tests to investigate *Leishmania* spp. infections in stray dogs from an area of intense visceral leishmaniasis transmission in Brazil. Rev. Bras. Parasitol. Vet. 30, e006621. 10.1590/S1984-29612021067 34406212

[16] Goto H, Lindoso JAL (2010) Current diagnosis and treatment of cutaneous and mucocutaneous leishmaniasis. Expert Rev Anti Infect Ther 8:419–433. 10.1586/eri.10.19 20377337

[17] Lachaud L, Marchergui-Hammami S, Chabbert E, Dereure J, Dedet JP, Bastien P (2002) Comparison of six PCR methods using peripheral blood for detection of canine visceral leishmaniasis. J Clin Microbiol 40:210–215. 10.1128/JCM.40.1.210-215.2002 PMC120090 11773118

[18] Lago J, Fraga D, Coelho L, de Jesus MS, Leite B, Werneck GL, Arruda S, Lago E, Carvalho EM, Bacellar O (2023) Dogs harbor *Leishmania braziliensis* and participate in the transmission cycle of human tegumentary leishmaniasis. Pathogens 12:981. 10.3390/pathogens12080981 PMC10458093 37623941

[19] Lima BS, Dantas-Torres F, de Carvalho MR, Marinho-Junior JF, de Almeida EL, Brito MEF, Gomes F, Brandão-Filho SP (2013) Small mammals as hosts of *Leishmania* spp. in a highly endemic area for zoonotic leishmaniasis in north-eastern Brazil. Trans R Soc Trop Med Hyg 107:592–597. 10.1093/trstmh/trt062 23868744

[20] Lima JTR, Gennari SM, Soares HS, Minervino AHH, Malheiros AF, Marques FS, Laurenti MD, Machado RZ, Marcili A, Labruna MB, Soares RM (2017) Serodiagnosis of visceral and cutaneous leishmaniasis in human and canine populations living in Indigenous Reserves in the Brazilian Amazon Region. Rev Soc Bras Med Trop 50:61–66. 10.1590/0037-8682-0377-2016 28327803

[21] Marinho RM, Fonteles RS, Vasconcelos GC, Azevêdo PCB, Moraes JLP, Rebêlo JMM (2008) Flebotomíneos (Diptera, Psychodidae) em reservas florestais da área metropolitana de São Luís, Maranhão, Brasil. Rev Bras Entomol 52:112–116. 10.1590/S0085-56262008000100019

[22] Marzochi MCA, Barbosa DS, Belo VS, de Araújo GR, da Silva AF, Pimentel MI, Marzochi KBF (2023) Visceral leishmaniasis in Brazil: scenarios and challenges for the surveillance and control. Rev Patol Trop 52:1–10. 10.5216/rpt.v52i1.74769

[23] Mendes CS, Coelho AB, Féres JG, Souza EC, Cunha DA (2016) Impacto das mudanças climáticas sobre a leishmaniose no Brasil. Cienc Saude Colet 21:263–272. 10.1590/1413-81232015211.03992015 26816183

[24] Mendes WS, Silva AAM, Trovão JR, Silva AR, Costa JML (2002) Expansão espacial da leishmaniose visceral americana em São Luís, Maranhão. Brasil Rev Soc Bras Med Trop 35:227–231. 10.1590/S0037-86822002000300005 12045815

[25] Menegatti JA, Dias AFLR (2024) Epidemiology of visceral leishmaniasis in municipalities of Mato Grosso and the performance of surveillance activities: an updated investigation. Rev Bras Parasitol Vet 33:e015623. 10.1590/S1984-29612024008 PMC10878697 38324884

[26] Nunes JB, Laurenti MD, Kanamura HY, Pereira AAC, Colombo FA, Marques MJ (2016) *Leishmania infantum* infection in dogs from the southern region of Minas Gerais State, Brazil. Rev Inst Med Trop São Paulo 58:75. 10.1590/S1678-9946201658075 PMC5096629 27828616

[27] Oliveira TMFS, Furuta PI, Carvalho D, Machado RZ (2008) A study of cross-reactivity in serum samples from dogs positive for *Leishmania* sp., *Babesia canis* and *Ehrlichia canis* in enzyme-linked immunosorbent assay and indirect fluorescent antibody test. Rev Bras Parasitol Vet 17:7–11. 10.1590/S1984-29612008000100002 18554433

[28] Otranto D, Paradies P, Sasanelli M, Spinelli R, Brandonisio O (2004) Rapid immunochromatographic test for serodiagnosis of canine leishmaniasis. J Clin Microbiol 42:2769–2770. 10.1128/JCM.42.6.2769-2770.2004 PMC427813 15184465

[29] Pacheco, T. dos A., Marcili, A., Costa, A.P. da, Witter, R., Melo, A.L.T., Vilas Boas, R., Chitarra, C.S., Dutra, V., Nakazato, L., Pacheco, R. de C., 2018. Genetic diversity and molecular survey of *Trypanosoma (Megatrypanum) theileri* in cattle in Brazil’s western Amazon region. Rev. Bras. Parasitol. Vet. 27, 579–583. 10.1590/S1984-296120180049 30133593

[30] Penha TA, Santos ACG, Rebêlo JMM, Moraes JLP, Guerra RMSNC (2013) Fauna de flebotomíneos (Diptera: Psychodidae) em área endêmica de leishmaniose visceral canina na região metropolitana de São Luís – MA, Brasil. Biotemas 26:121–127. 10.5007/2175-7925.2013v26n2p121

[31] Pennisi MG, Cardoso L, Baneth G, Bourdeau P, Koutinas A, Miró G, Oliva G, Solano-Gallego L (2015) LeishVet update and recommendations on feline leishmaniosis. Parasit Vectors 8:302. 10.1186/s13071-015-0909-z PMC4462189 26041555

[32] Persichetti MF, Solano-Gallego L, Vullo A, Masucci M, Marty P, Delaunay P, Vitale F, Pennisi MG (2017) Diagnostic performance of ELISA, IFAT and Western blot for the detection of anti-*Leishmania infantum* antibodies in cats using a Bayesian analysis without a gold standard. Parasites Vectors 10:119. 10.1186/s13071-017-2046-3 PMC5346856 28285598

[33] Pineda V, Calzada JE, Montilla S, Rodríguez I, Howard E, Torres AI, Vasquez V, Reina A, Saldaña A, González K (2025) Molecular detection of *Leishmania (V.) braziliensis* and *Leishmania (M.) martiniquensis* infecting domestic animals from Panama, Central America. Animals (Basel) 15:2677. 10.3390/ani15182677 PMC12466877 41007920

[34] Rangel EF, Lainson R (2009) Proven and putative vectors of American cutaneous leishmaniasis in Brazil: aspects of their biology and vectorial competence. Mem Inst Oswaldo Cruz 104:937–954. 10.1590/S0074-02762009000700001 20027458

[35] Rebêlo JMM, Araújo JAC, Carvalho ML, Barros VLL, Silva FS, Oliveira ST (1999) Flebótomos (Diptera, Phlebotominae) da Ilha de São Luís, zona do Golfão Maranhense. Brasil Rev Soc Bras Med Trop 32:247–253. 10.1590/S0037-86821999000300005 10380563

[36] Rêgo FD, Soares RP (2021) *Lutzomyia longipalpis*: an update on this sand fly vector. An Acad Bras Cienc 93:e20200254. 10.1590/0001-3765202120200254 33950136

[37] Rocha AVVO, Moreno BFS, Cabral AD, Louzeiro NM, Miranda LM, Santos VMBD, Costa FB, Nogueira RMS, Marcili A, Sperança MA, Costa APD (2019) Diagnosis and epidemiology of *Leishmania infantum* in domestic cats in an endemic area of the Amazon region, Brazil. Vet Parasitol 273:80–85. 10.1016/j.vetpar.2019.08.007 31446257

[38] Roque ALR, Jansen AM (2014) Wild and synanthropic reservoirs of *Leishmania* species in the Americas. Int J Parasitol Parasites Wildl 3:251–262. 10.1016/j.ijppaw.2014.08.004 PMC4241529 25426421

[39] da Sanches LC, Martini CC, Nakamura AA, Santiago MEB, Lima BD, Lima VMF (2016) Natural canine infection by *Leishmania infantum* and *Leishmania amazonensis* and their implications for disease control. Rev Bras Parasitol Vet 25:465–469. 10.1590/S1984-29612016071 27925065

[40] Santos LEN, Costa MCL (2020) O planejamento urbano em São Luís pela legislação de zoneamento, parcelamento, uso e ocupação do solo: limites ao direito à cidade pelo processo de produção do espaço. Caminhos de Geografia 21:36–50. 10.14393/RCG217447447

[41] Schönian G, Nasereddin A, Dinse N, Schweynoch C, Schallig HD, Presber W, Jaffe CL (2003) PCR diagnosis and characterization of *Leishmania* in local and imported clinical samples. Diagn Microbiol Infect Dis 47:349–358. 10.1016/S0732-8893(03)00093-2 12967749

[42] da Silva AR, Tauil PL, Cavalcante MNS, Medeiros MN, Pires BN, do R. Gonçalves EdaG (2008) Situação epidemiológica da leishmaniose visceral, na Ilha de São Luís, Estado do Maranhão. Rev Soc Bras Med Trop 41:358–364. 10.1590/S0037-86822008000400007 18853007

[43] Silveira APS, Vieira VBD, Batalini LS, Carmo SB, Friozi E, Arruda EJ, da C. Lima Junior MS, Neitzke-Abreu HC (2018) PCR sensitivity of peripheral blood of dogs co-infected with *Leishmania* spp. and *Ehrlichia* spp. in endemic area of Brazil. Rev Soc Bras Med Trop 51:843–847. 10.1590/0037-8682-0040-2018 30517541

[44] Silveira FT, Lainson R, Corbett CEP (2004) Clinical and immunopathological spectrum of American cutaneous leishmaniasis with special reference to the disease in Amazonian Brazil: a review. Mem Inst Oswaldo Cruz 99:239–251. 10.1590/S0074-02762004000300001 15273794

[45] Solano-Gallego L, Koutinas A, Miró G, Cardoso L, Pennisi MG, Ferrer L, Bourdeau P, Oliva G, Baneth G (2009) Directions for the diagnosis, clinical staging, treatment and prevention of canine leishmaniosis. Vet Parasitol 165:1–18. 10.1016/j.vetpar.2009.05.022 19559536

[46] Solano-Gallego L, Miró G, Koutinas A, Cardoso L, Pennisi MG, Ferrer L, Bourdeau P, Oliva G, Baneth G (2011) LeishVet guidelines for the practical management of canine leishmaniosis. Parasit Vectors 4:86. 10.1186/1756-3305-4-86 PMC3125381 21599936

[47] Souza AI, Barros EMS, Ishikawa E, Ilha IMN, Marin GRB, Nunes VLB (2005) Feline leishmaniasis due to *Leishmania (Leishmania) amazonensis* in Mato Grosso do Sul State, Brazil. Vet Parasitol 128:41–45. 10.1016/j.vetpar.2004.11.020 15725531

[48] Souza AI, Nunes VLB, Borralho VM, Ishikawa EAY (2009) Domestic feline cutaneous leishmaniasis in the municipality of Ribas do Rio Pardo, Mato Grosso do Sul state, Brazil: a case report. J Venom Anim Toxins Incl Trop Dis 15:359–365. 10.1590/S1678-91992009000200017

[49] Thrusfield M (2004) Epidemiologia Veterinária, 2nd ed. Roca, São Paulo

[50] Valero NNH, Uriarte M (2020) Environmental and socioeconomic risk factors associated with visceral and cutaneous leishmaniasis: a systematic review. Parasitol Res 119:365–384. 10.1007/s00436-019-06575-5 31897789

[51] Van Dijk NJ, Hagos DG, Huggins DM, Carrillo E, Ajala S, Chicharro C et al (2024) Simplified molecular diagnosis of visceral leishmaniasis: laboratory evaluation of miniature direct-on-blood PCR nucleic acid lateral flow immunoassay. PLoS Negl Trop Dis 18:e0011637. 10.1371/journal.pntd.0011637 PMC11075898 38713648

[52] Volf P, Myskova J (2007) Sand flies and *Leishmania*: specific versus permissive vectors. Trends Parasitol 23:91–92. 10.1016/j.pt.2006.12.010 PMC2839922 17207663

